# Knockdown of RNA-binding protein IMP3 suppresses oral squamous cell carcinoma proliferation by destabilizing E2F5 transcript

**DOI:** 10.18632/aging.205466

**Published:** 2024-01-24

**Authors:** Zhenzhen Wang, Huahua Zhang, Fang Li, Chen Huang

**Affiliations:** 1 Key Laboratory of Shaanxi Province for Craniofacial Precision Medicine Research, College of Stomatology, Xi'an Jiaotong University, Xi'an, Shaanxi, China; 2 Department of Cell Biology and Genetics/Key Laboratory of Environment and Genes Related to Diseases, School of Basic Medical Sciences, Xi’an Jiaotong University Health Science Center, Xi’an, Shaanxi, China; 3 Medical Research and Experimental Center, Medical College, Yan’an University, Yan’an, Shaanxi, China

**Keywords:** oral squamous cell carcinoma, IMP3, E2F5, cell proliferation

## Abstract

The expression level of RNA-binding proteins (RBPs) is dysregulated in oral squamous cell carcinoma (OSCC) and other types of cancer. Among the RBPs, IMP3 is involved in the progression of OSCC. However, the regulation of mRNA fate by IMP3 in OSCC remains less understood. We analyzed the expression level of IMP3 and E2F5 in OSCC tissues and cell lines by immunohistochemistry, qRT-PCR and Western blot. Subsequently, to further investigate the effect of IMP3 on E2F5 expression, we used siRNAs to silence IMP3 expression in OSCC cell lines SCC-25 and SCC-4. The binding site of E2F5 mRNA and IMP3 was confirmed by RNA immunoprecipitation (RIP). Finally, the function of IMP3 and E2F5 was investigated *in viro* and in xenograft mouse models. Here we report a positive correlation between IMP3 and E2F5 expression in OSCC, which are involved in cell proliferation and cell cycle. Mechanistically, E2F5 mRNA is bound by IMP3 protein, and silencing it leads to a shortened mRNA half-life and reduced protein expression. Also, knockdown of IMP3 inhibited allograft tumor progression *in vivo*. These studies reveal the molecular mechanism by which IMP3 regulates E2F5 mRNA stability and identify IMP3/E2F5 as a potential therapeutic target in OSCC.

## INTRODUCTION

Oral squamous cell carcinoma (OSCC) is one of the most common malignant tumors, and the number of cases globally is ~400,000 per year, ranking it first among head and neck cancers. Moreover, OSCC has a poor prognosis, with a 5-year survival rate of less than 50% in most parts of the world [1, 2]. The pathogenesis of OSCC is complex. At present, many scholars believe that the disruption of the balance between oncogene activation and tumor suppressor gene inhibition in cells may be one of the causes of OSCC. Due to the high degree of malignancy, rapid progression, and easy recurrence of OSCC, most patients are at an advanced stage when they are diagnosed, which brings challenges to treatment [3, 4]. Therefore, the importance of elucidating the molecular mechanisms, occurrence, and development of OSCC along with identifying diagnostic markers and therapeutic targets is needed.

Previous studies on the molecular mechanism of OSCC development mainly focus on epigenetic modification [5], gene mutation [6], and abnormal regulation of non-coding RNA [7, 8]. In recent years, research on RNA-binding proteins (RBPs) has become an important direction in the field of tumor pathogenesis and progression [9]. Functional studies over the last decades revealed that ADAR1, DDX3, ESRP1 IMPs and LIN28B associated with migration, metastasis and invasion in OSCC [10–14]. IMPs are a class of RBPs, including IMP1, 2, and 3. In recent years, the molecular regulation of IMP3 has attracted particular attention. Its mechanism of action is reported to include target mRNA binding to form RNA complexes that stabilize the mRNA and inhibit its degradation, interact with RISC to inhibit miRNA attacking target mRNA, recognize m6A-modified mRNA and regulate its expression [15, 16]. Together, IMP3 regulates the post-transcriptional processing, transport, and translation of RNA and affects the fate of mRNA.

IMP3 was identified due to its high abundance in pancreatic cancer tissue. Previous studies have shown that dysregulation of IMP3 is associated with the growth, migration, adhesion, and energy metabolism of cancer cells. It also modulates the occurrence and development of many human diseases such as diabetes and malignant tumors [17–19]. Numerous studies have suggested that IMP3 expression to be upregulated in OSCC. Nevertheless, several studies proved that IMP3 as a predictor of metastasis and its expression was shown to correlate with an overall poor prognosis in OSCC [13, 20, 21]. Hwang reported a key target of IMP3 in OSCC seems to be podoplanin (PDPN). PDPN is also specifically expressed at the invasive front of tumors and IMP3 regulates the PDPN expression by binding to the 3’UTR of the PDPN mRNA, and then stabilizes the transcript [22, 23]. However, additional studies are required to investigate IMP3 functions in binding and regulating transcripts in OSCC progression.

In this study, we identified oncogenic roles of IMP3 in OSCC *in vitro* and *in vivo*. IMP3 was upregulated and frequently observed in OSCC. Further investigation demonstrated that knockdown of IMP3 suppressed OSCC cell proliferation by targeting E2F5 mRNA. Taken together, our results indicate a molecular mechanism by which IMP3 regulates E2F5 mRNA transcript, which could be a therapeutic target for OSCC.

## MATERIALS AND METHODS

### Human tissue samples

OSCC tissues and their paired adjacent normal tissues (from 40 patients) were obtained from the College of Stomatology, Xi’an Jiaotong University. Informed consent was obtained from each enrolled patient, and the study was approved by the Ethics Committee of Xi’an Jiaotong University (approval number 2020393).

### Cell culture and transfection

Human OSCC cell lines SCC-25, SCC-4, and HOK cells (human oral keratinocytes) were purchased from the Cell Bank (Shanghai Institute of Biochemistry and Cell Biology, China). All cells were maintained in DMEM high glucose medium containing 10% FBS (Gibco, USA) and 1% antibiotics (penicillin/streptomycin). Transient transfection of siRNA and their respective negative control RNAs was performed using Polyplus transfection kit (Jetprime, France) following the manufacturer’s instructions.

### qRT-PCR

Total RNA was collected from cells and extracted with TRIzol Reagent (Invitrogen, USA). cDNA was made using PrimeScript^™^ RT reagent kit (Takara, Japan). qRT-PCR was carried out using the SYBR Premix Ex Taq II Kit (Takara, Japan) on IQ5 Multicolor PCR Detection System (Bio-Rad, USA). β-actin was used as an internal reference and 2^-ΔΔCt^ method was used to determine relative abundance. Three replicates per reaction were performed for qRT-PCR. The primers used are as follows: β-actin forward 5’-CCAGAGGCGTACAGGGATAG-3’; reverse 5’- CCAACCGCGAGAAGATGA-3’; IMP3 forward 5’-CCTGGTGAAGACTGGCTACG-3’; reverse 5’- ATCCAGCACCTCCCACTGTA-3’; E2F5 forward 5’-TCAGGCACCTTCTGGTACAC-3’; reverse 5’-GGGCTTAGATGAACTCGACTC-3’.

### Western blotting assays

Total cell lysates were lysed in RIPA buffer (Roche, Switzerland), and protein concentration was determined using a BCA protein assay kit (Thermo Fisher Scientific, USA). Equal amounts of proteins were separated on 10% SDS-PAGE gels and transferred to PVDF membranes. The membranes were blocked with 5% non-fat milk and then incubated with primary antibodies (anti-IMP3, Abcam, USA; anti-E2F5, Abcam, USA; anti-CDK4 and anti-CyclinD1, 1: 2000, Cell Signaling Technology, USA) at 4° C overnight. Following a wash, membranes were incubated with secondary antibodies for 1 h, washed, and developed using ECL (Pierce, USA).

### Immunohistochemistry (IHC)

Tissue sections were deparaffinized in xylene and hydrated with an alcohol gradient. Antigen retrieval and endogenous peroxidase blocking were performed sequentially. Tissues were probed with polyclonal rabbit anti-IMP3 (diluted 1:100) and anti-E2F5 (diluted 1:100) at 4° C overnight, washed, and next incubated with secondary antibody (1:5000) for 1 h at room temperature. Tissues were stained with 3,3′-diaminobenzidine kit (DAB, China) and hematoxylin for histological examination. Tissue sections were fixed and images captured under an inverted microscope.

### CCK8 assay

CCK8 assay kit (7Sea Biotech, China) was employed to study the cell proliferation of SCC-25 and SCC-4 cells according to the manufacturer' instructions. Briefly, the transfected cells were seeded into 96-well plated with 3000 cells/well. CCK8 solution (10 μl) was added into each well after 24, 48 and 72 h, then the 96-well plates were placed in a humidified incubator for additional incubation of 4 h. The OD value was obtained at 450 nm with FLUOstar OPTIMA (BMG, Germany).

### Cell cycle assay

OSCC cells were seeded in 6-well plates (1 × 10^6^ cells/well) and transfected with siRNA. After 48 hours, the cells were collected and fixed in 70% ethanol and stored at 4° C overnight. The next day the samples were stained with 10 μg/mL propidium iodide according to the manufacturer’s instructions (Sigma, USA) and incubated for 1 hour at room temperature in the dark. Cells were then analyzed by Flow Cytometer (BD Biosciences, USA).

### RNA stability assay

The cells were seeded in 6-well plates and treated with 5 μg/mL actinomycin D. The cells were collected at 3 h, 6 h, and 9 h post-treatment. The total RNA was extracted for RT-qPCR as described above. The degradation rate of the mRNAs was estimated following published protocols [24].

### RNA immunoprecipitation (RIP) assay

For the RIP assay, the Magna RIP™ Kit (No. 17-701, Millipore, USA) was performed according to the manufacturer’s instructions. OSCC cells were lysed in RIP lysis buffer supplemented with proteinase inhibitors and RNAse inhibitor and then incubated with anti-IMP3 or IgG-coupled protein beads for 6 h to overnight at 4° C. The next day after stringently washing the beads with washing buffer, the immunoprecipitated RNA was purified and analyzed by qRT-PCR.

### Mouse xenograft studies

Stable IMP3-deficient SCC-25 cells were injected into six-week-old, female, nude mice (n = 6/group), for tumor engraftment. Tumors were measured every 5 days for 30 days. Four weeks after tumor inoculation, mice were euthanized by cervical dislocation under anesthesia. The tumors were excised and analyzed. All the experimental protocols were approved by the Institutional Animal Care and Use Committee of Xi'an Jiaotong University.

### Bioinformatics analysis

The gene expression data were obtained at The Cancer Genome Atlas website for OSCC projects (TCGA, https://tcga-data.nci.nih.gov/tcga/).

### Statistical analysis

All the above experiments were repeated three times. Differences between groups were analyzed using Student’s t-test or one-way ANOVA; also to determine if the quantitative data showed normal distribution. If data were not normally distributed, the Wilcoxon-Mann-Whitney test was performed. All statistical analyses were carried out using SPSS 18.0 (IBM, USA). P < 0.05 was considered significant, and data are presented as the mean ± Standard error of the mean (SEM).

## RESULTS

### IMP3 is overexpressed in OSCC

**Figure 1:**
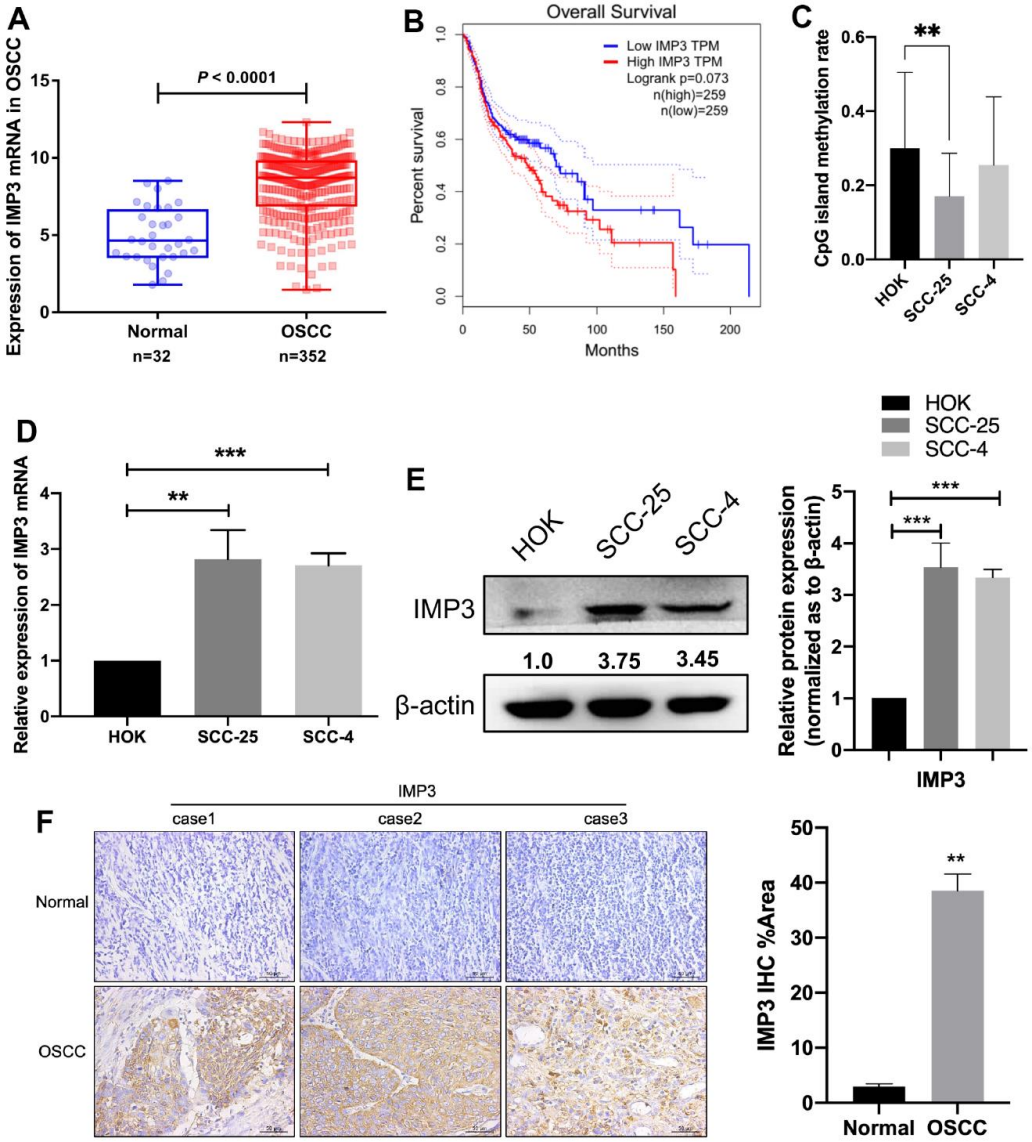
**Overexpression of IMP3 in human OSCC tissues and cell lines.** (**A**) TCGA analysis showed that IMP3 mRNA levels were increased in the OSCC tissues (n=352) compared with the normal tissues (n=32). (**B**) Kaplan-Meier analysis was performed on OSCC patient survival with the GEPIA database according to high or low expression of IMP3. (**C**) Methylation sequencing results showed that the high expression of IMP3 in oral squamous cell carcinoma was probably related to CpG island methylation modification. Expression level of IMP3 mRNA (**D**) and protein (**E**) in OSCC cell lines (SCC-25 and SCC-4) in comparison with HOK cell line. (**F**) The IHC score of IMP3 was markedly higher in the OSCC tissues than in the adjacent normal tissues (n=40). Magnification ×40. The scale bar indicates 50 μm. Relative expression levels were calculated using the image J software (n = 3). *P*≤ 0.05 was considered to be statistically significant, **P <0.01, ***P< 0.001.

To identify the roles of IMP3 in OSCC, we examined the expression of IMP3. From TCGA database, we found that IMP3 is overexpressed in OSCC compared to normal tissue (Figure 1A). Western blot and qRT-PCR analysis showed that IMP3 expression was enriched in OSCC cell lines (SCC-25 and SCC-4) compared to HOK cells (Figure 1D, 1E). Meanwhile, methylation sequencing results showed that the high expression of IMP3 in oral squamous cell carcinoma was probably related to CpG island methylation modification (Figure 1C). Patients with high IMP3 expression had markedly lower overall survival rates compared to those with low IMP3 expression (Figure 1B). Next, we measured the expression of IMP3 in 40 paired patient samples of OSCC tissue and adjacent normal tissue with IHC (Figure 1F). Consistent with *in vitro* results, expression of IMP3 was significantly increased in OSCC, and higher expression of IMP3 was observed in the cytoplasm. The IMP3 protein expression increased in 3 pairs of OSCC tissues compared with normal tissues (Figure 1F). Collectively, these findings demonstrate that IMP3 expression is overregulated in OSCC.

### Knockdown of IMP3 inhibits OSCC cell growth

**Figure 2:**
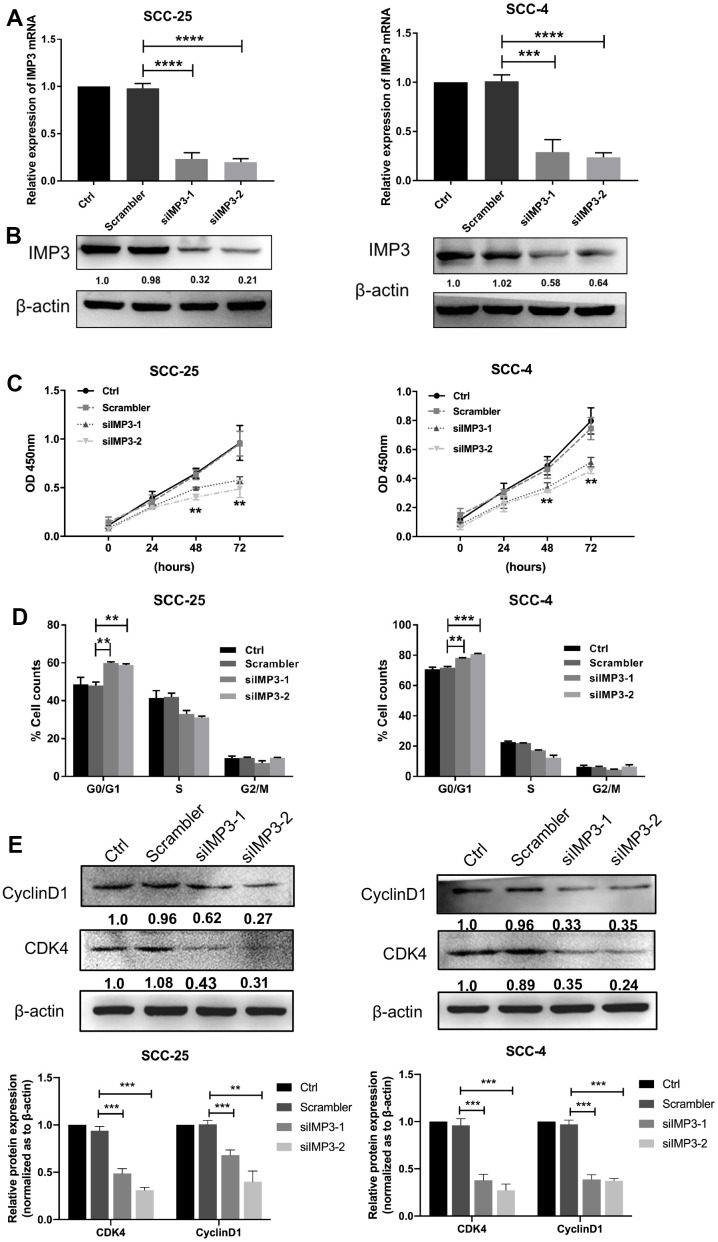
**Knockdown of IMP3 inhibits OSCC proliferation.** The expression of IMP3 in SCC-25 and SCC-4 cells transfected with IMP3 siRNA was determined by qRT-PCR (**A**) and Western blot (**B**). (**C**) Cell growth in OSCC cells transfected with siIMP3 as measured by CCK8 assay(n=5). (**D**) Effects of suppression of IMP3 on the cell cycle progression of SCC-25 and SCC-4 cells measured by flow cytometric analysis. (**E**) Expression analysis for cell cycles regulators Cyclin D1 and CDK4 was performed by Western blot. Relative expression levels were calculated using the image J software (n = 3). *P* ≤ 0.05 was considered to be statistically significant, **P < 0.01, ***P < 0.001, ****P < 0.0001.

To further investigate the functional roles of IMP3 in OSCC, we used siRNA depletion of IMP3 in SCC-25 and SCC-4 cells to reduce the mRNA and protein levels of IMP3 to 20~30% tested by qRT-PCR and Western blot (Figure 2A, 2B). Then CCK8 and cell cycle assays were performed on OSCC cells. In the CCK8 assay, knockdown of IMP3 significantly inhibited cell proliferation compared to siRNA control (Figure 2C). In the cell cycle assay, suppression of IMP3 in OSCC cells markedly decreased cell viability and led to a decrease in the number of cells in the G1, owing to the decreased levels of CDK4 and Cyclin D1 (Figure 2D, 2E). Taken together, these findings suggest that IMP3 drives OSCC cell growth *in vitro*.

### IMP3 positively regulates E2F5

**Figure 3:**
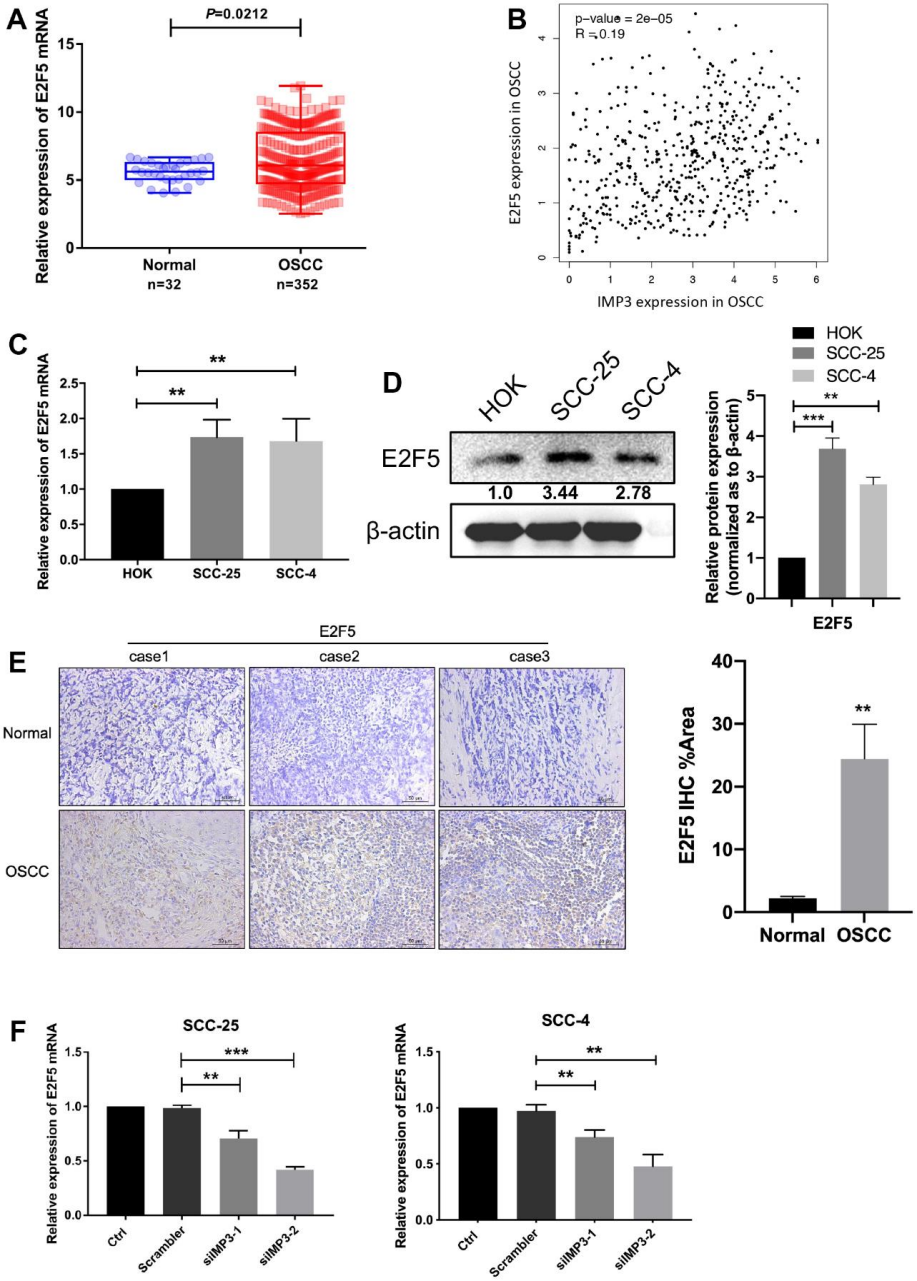
**IMP3 positively regulated E2F5.** (**A**) TCGA analysis showed that E2F5 mRNA levels were increased in the OSCC tissues (n=352) compared with the normal tissues (n=32). (**B**) Pearson correlation analysis of IMP3 and E2F5 expression in OSCC showed that IMP3 is positively correlated with E2F5. (**C**, **D**) qRT-PCR and Western blot assay showed that E2F5 was upregulated in OSCC cell lines (SCC-25 and SCC-4) in comparison with HOK cell line. (**E**) Representative images of IHC staining of E2F5 protein in OSCC tissues (n=40). Magnification ×40. The scale bar indicates 50 μm. (**F**) Quantification of E2F5 transcripts in SCC-25 and SCC-4 after transfection with siIMP3. Relative expression levels were calculated using the image J software (n = 3). *P* ≤ 0.05 was considered to be statistically significant, **P < 0.01, ***P < 0.001.

Even though IMP3 is overexpressed in OSCC and knockdown of IMP3 suppresses cell growth, the molecular mechanisms of IMP3 regulation remained unknown. Using TCGA database analysis, we identified that E2F5 was upregulated in OSCC tissue and had a positive correlation with IMP3 (Figure 3A, 3B). Examination of the level of E2F5 in OSCC tissue and cell lines, as showed in Figure 3C, 3D, E2F5 was distinctly overexpressed in SCC-25 and SCC-4 cell lines when compared to that in HOK cell. Besides, E2F5 was abundant in OSCC tissue in comparison to normal tissues as demonstrated by IHC staining (Figure 3E). Moreover, knockdown of IMP3 significantly down-regulated E2F5 RNA levels in SCC-25 and SCC-4 cell lines (Figure 3F). Thus, all these data imply that IMP3 positively regulates E2F5 in OSCC.

### IMP3 stabilizes E2F5 mRNA

**Figure 4:**
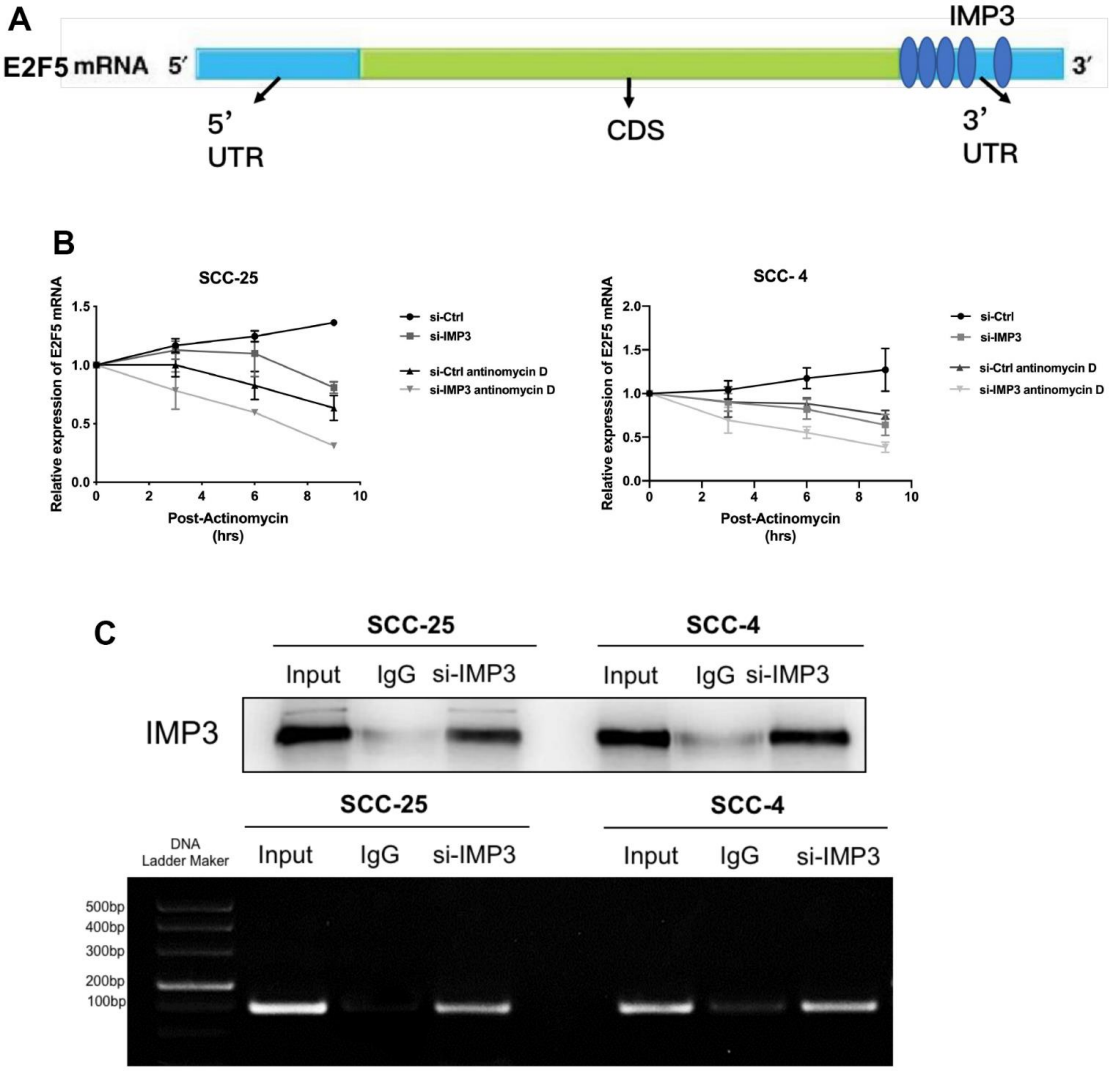
**E2F5 is a direct target of IMP3.** (**A**) The schematic image of the binding sites of IMP3 in the TIMM44 mRNA. (**B**) Analysis of the mRNA half-lives of E2F5 expression in the SCC-25 and SCC-4 cells suppression of IMP3. (**C**) RIP analysis of IMP3 binding to E2F5 mRNA in OSCC cells. Nonspecific rabbit IgG was used as a negative control. Input was used as a positive control.

Since IMP3 is an RBP and can promote the stability of bound RNA, its effect may be exerted on E2F5 mRNA. Therefore, we analyzed the function of IMP3 on E2F5 mRNA stability in OSCC cells. Upon analyzing the E2F5 mRNA sequence, we identified IMP3 binding motifs (Figure 4A). We investigated the stability of E2F5 mRNA at different times in the presence of actinomycin D (Figure 4B). Suppression of IMP3 significantly decreased the stability and half-life of E2F5 mRNA. To confirm the predicted binding sites, we performed RIP assays. As shown in (Figure 4C), E2F5 mRNA was enriched in IMP3 IP samples compared to the IgG IP samples, indicating that IMP3 specifically binds to E2F5 mRNA. Taken together, these findings demonstrate that E2F5 mRNA is a direct target of IMP3.

### Suppression of E2F5 inhibits the OSCC cell proliferation

**Figure 5:**
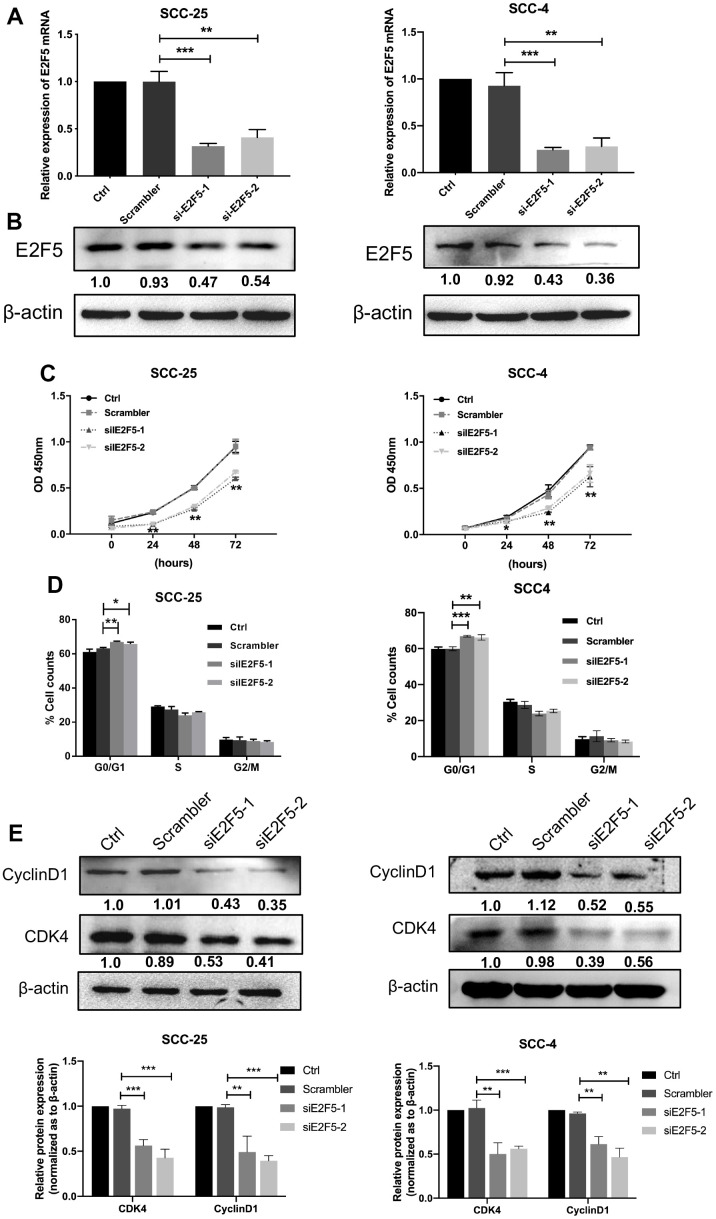
**Effects of E2F5 inhibition on OSCC cell growth.** The expression of E2F5 was analyzed with qRT-PCR (**A**) and Western blot (**B**) after transfection with siE2F5 and its control. (**C**) Cell viability of SCC-25 and SCC-4 cells transfected with siE2F5 was determined by the CCK8 assay (n=5). (**D**) Effects of suppression of E2F5 on the cell cycle progression of OSCC cells measured by flow cytometric analysis. (**E**) Expression analysis for cell cycles regulators Cyclin D1 and CDK4 was performed by Western blot. Relative expression levels were calculated using the image J software (n = 3). *P* ≤ 0.05 was considered to be statistically significant, *P < 0.05, **P < 0.01, ***P < 0.001.

Given that the expression of E2F5 was strongly correlated with IMP3, we next studied the functions of E2F5 in OSCC. Transient silencing of E2F5 in SCC-25 and SCC-4 with siRNA significantly down-regulated the expression of E2F5 as determined by qRT-PCR and Western blot (Figure 5A, 5B). Suppression of E2F5 inhibited the proliferation of OSCC cells as determined by CCK8 assay (Figure 5C). Additionally, cell cycle assays showed that inhibition of E2F5 in SCC-25 and SCC-4 cells resulted in decreased cell numbers in G1 phase due to reduced levels of CDK4 and Cyclin D1 (Figure 5D, 5E). Collectively, these data demonstrate that downregulation of E2F5 suppresses the growth of OSCC cell which shared similar effects of silencing of IMP3.

**Figure 6:**
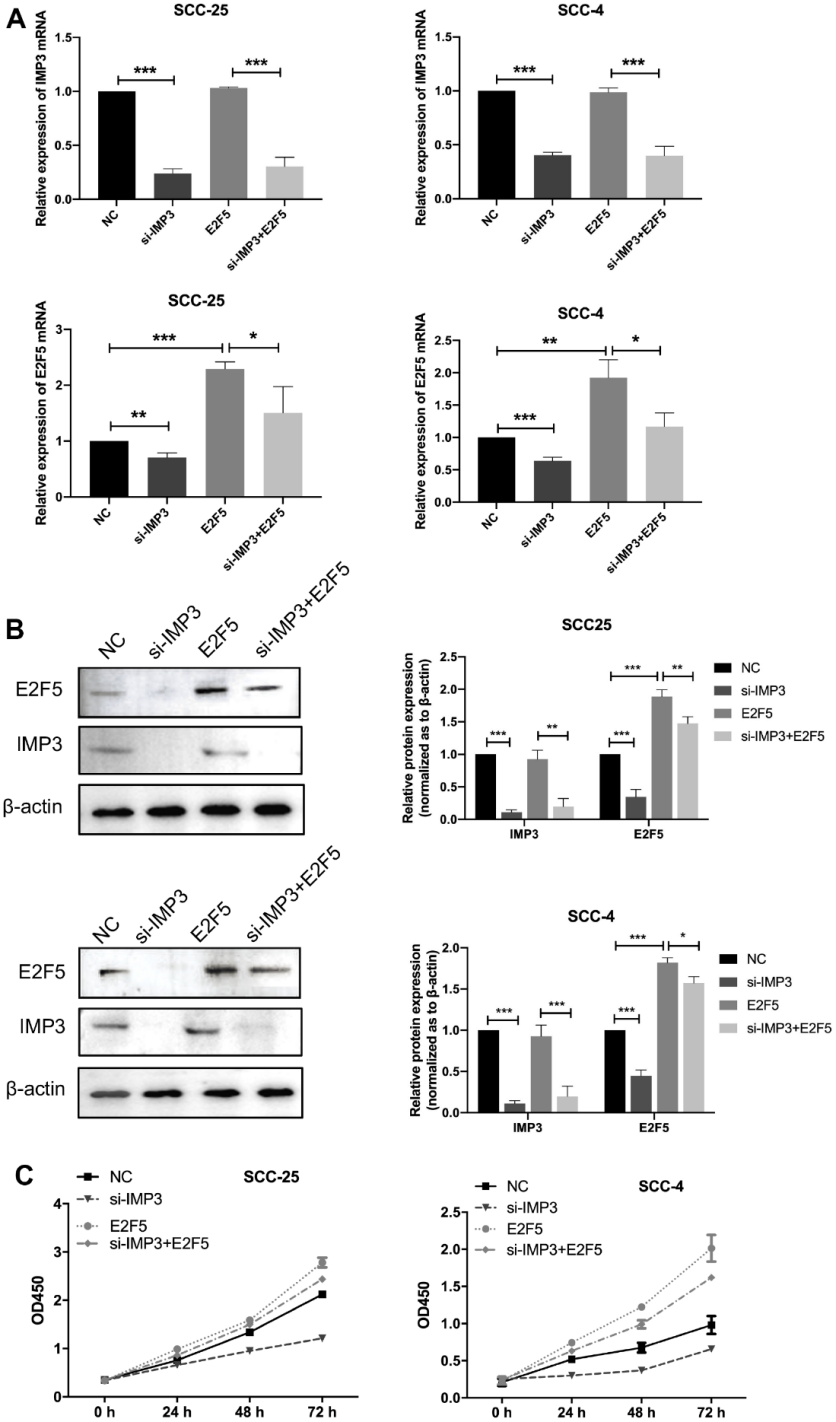
**Knockdown of IMP3 reverses E2F5-induced cellular phenotypes in OSCC cells.** (**A**, **B**) qRT-PCR and Western blot were performed to determine the impact of OSCC cells treated with E2F5 plus siIMP3 expression vectors or related negative control. (**C**) The growth of OSCC cells were detected using CCK8 assay. Relative expression levels were calculated using the image J software (n = 6). *P* ≤ 0.05 was considered to be statistically significant, *P < 0.05, **P < 0.01, ***P < 0.001.

To further explore that IMP3 regulated tumor progression through regulating E2F5, SCC-25 and SCC-4 cells were transfected siIMP3 alone or cotransfected with siIMP3 plus E2F5 vector. As showed in Figure 6A, 6B, knockdown of IMP3 partly reversed the cells from the effects of E2F5 on regulation of E2F5 expression. Moreover, CCK8 assay suggested that silencing of IMP3 strongly suppressed the cell growth of OSCC cell. Nevertheless, E2F5 reversed the inhibitory effects of siIMP3 (Figure 6C). All these findings demonstrate that IMP3 regulates the progression of OSCC by regulating E2F5.

### Ectopic expression of IMP3 inhibits xenograft tumor growth

**Figure 7:**
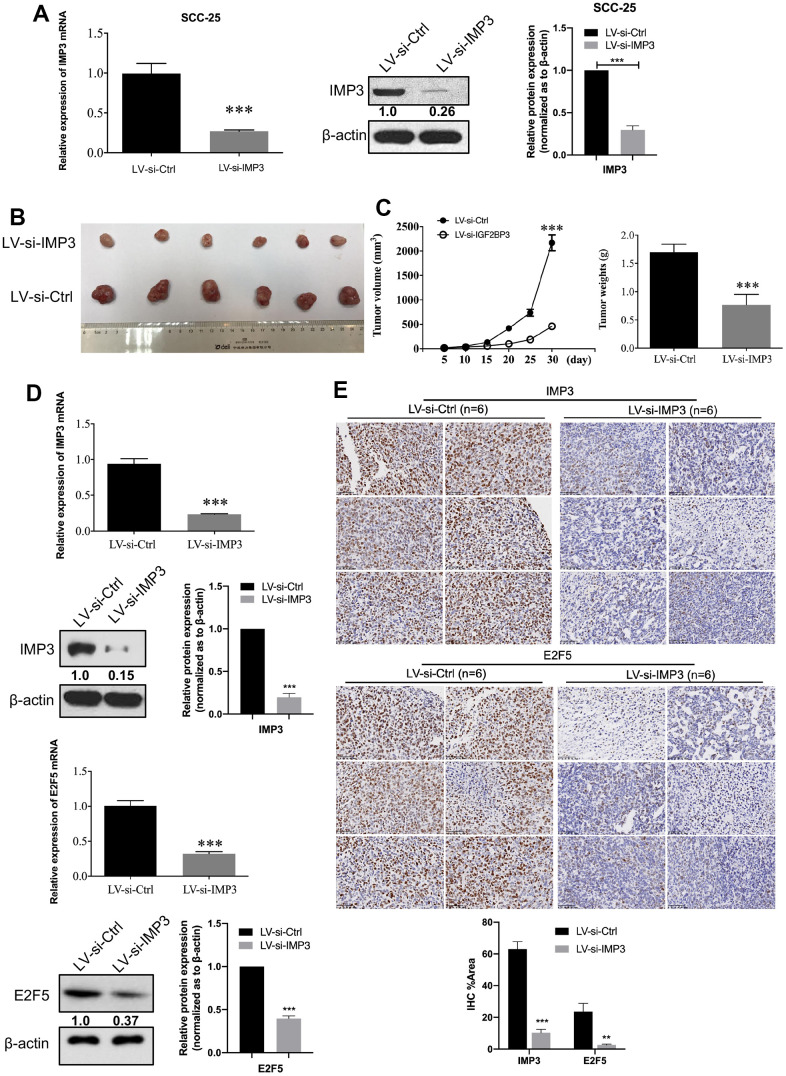
**IMP3-depleted inhibits OSCC cell proliferation.** (**A**) Establishment of IMP3-depleted SCC-25 cells. The expression of IMP3 was analyzed with qRT-PCR and Western blot. Relative expression levels were calculated using the image J software (n = 3). (**B**) Stable IMP3-depleted SCC-25 cells and control cells were subcutaneously injected into nude mice. A representative image showing tumors from mice transplanted with SCC-25 cells with or without IMP3 inhibition. (**C**) The tumor growth and the tumor weights from each group were statistically analyzed (n=6). (**D**) qRT-PCR and Western blot analysis showed that IMP3 and E2F5 levels were increased in the tissues compared with the control tissues. (**E**) IHC staining of IMP3 (up) and E2F5 (down) in tumor tissues from mice implanted with SCC-25-LV-si-IMP3 or SCC-25-LV-si-Ctrl cells. Magnification ×40. Scale bar, 50 μm. Relative expression levels were calculated using the image J software (n = 6). *P* ≤ 0.05 was considered to be statistically significant, *P < 0.05, **P < 0.01, ***P < 0.001.

Since knockdown of IMP3 expression inhibited the malignant properties of OSCC cells *in vitro*, we also investigated the effect of IMP3 on the tumorigenicity in a xenograft model. SCC-25 cells stably transfected with LV-si-IMP3 or LV-si-Ctrl were injected subcutaneously into nude mice (Figure 7A). Tumor sizes were measured using a caliper every 5 days. After 30 days, the mice were euthanized, and tumor weights were measured. The results demonstrated that the tumor growth and tumor weight were significantly decreased in IMP3-knockdown cells as compared to controls (Figure 7B, 7C). Furthermore, qRT-PCR, Western blot, and IHC showed that the expression of IMP3 and E2F5 was significantly decreased in LV-si-IMP3 injected subcutaneous xenografts as compared to LV-si-Ctrl (Figure 7D, 7E).

## DISCUSSION

In the current study, we found that high IMP3 expression correlated with poor outcomes in OSCC patients. Also, knocking-down IMP3 in OSCC cell lines strongly decreased cell proliferation. These results suggest that IMP3 is a potential therapeutic target for inhibiting OSCC progression.

In recent years, the molecular regulation mechanism of IMP3 in cells has received particular attention. Previous studies showed that the expression of IMP3 is highly correlated with unfavorable prognosis in OSCC patients and that IMP3 promotes OSCC cells malignant progression [21, 25]. However, all studies relied on a non-paralogue-specific antibody and some results have to be considered with great caution. Although the detailed mechanisms that drive IMP3 expression in OSCC remain to be determined, a recent study identified epidermal growth factor (EGF) as an inducer of IMP3. In this study, we also found that IMP3 was up-regulated in OSCC tissues and cell lines (SCC-25 and SCC-4). Moreover, silencing of IMP3 significantly inhibited the growth of SCC-25 and SCC-4 cells. However, the mechanism by which IMP3 modulates cell viability in OSCC remained largely unknown.

IMP3 has been reported to bind to 3’-UTR of target mRNA and stabilize them, which is one of the mechanisms it is implicated in for cancer [26, 27]. By searching the TCGA database, we found that E2F5 was up-regulated in OSCC tissue and had a positive correlation with IMP3. E2F5 belongs to the E2F transcription factor family which is involved in cell growth, apoptosis, differentiation and cell cycle in cancers [28]. Previous studies demonstrate that aberrant expression of E2F5 is associated with the malignancy of ovarian cancer, gastric cancer, lung cancer, and hepatocellular carcinoma [29–32]. Ping Zhou recently reported that identification of E2F transcription factor 7 as a novel potential biomarker for oral squamous cell carcinoma [33]. Nevertheless, there are few reports about the impacts of E2Fs on OSCC, and the related mechanism has not been clarified, and needs further study. Thus, we wanted to know whether the increased proliferation of OSCC cells induced by IMP3 is mediated by E2F5.

By intersecting the results from RIP and RNA stability assays, we found that E2F5 is a direct target of IMP3 in SCC-25 and SCC-4 cell. Also, suppression of E2F5 significantly inhibited cell growth of OSCC. *In vivo* experiments confirmed that knockdown of IMP3 expression could significantly inhibit the growth of subcutaneous tumors in nude mice. Also, IHC showed that the inhibition of IMP3 expression suppressed the expression of E2F5. Therefore, our research revealed that IMP3 promotes E2F5 mRNA stability, which may play a significant role in the proliferation of OSCC.

In conclusion, our results demonstrate that IMP3 has an important role in driving E2F5 expression and the proliferation of OSCC, which will foster the development of therapies aimed at treating OSCC.
